# High-throughput sequencing reveals novel microRNAs in the Bovine Leukemia Virus (BLV)-induced ovine model of leukemia

**DOI:** 10.1186/1742-4690-8-S1-A15

**Published:** 2011-06-06

**Authors:** Nicolas Rosewick, Céline dehouck, Yvette Cleuter, Florian Caiment, Michel Georges, Philippe Martiat, Arsène Burny, Anne Van den Broeke

**Affiliations:** 1 Laboratory of Experimental Hematology, Institut Jules Bordet, Université Libre de Bruxelles (ULB), Brussels, 1000, Belgium; 2 Unit of Animal Genomics, GIGA-R, Université de Liège (ULg), Liège, 4100, Belgium

## 

Bovine Leukemia Virus (BLV), a delta-retrovirus related to humanT-cell leukemia virus-1 (HTLV-1), is associated with the development of B-cell leukemia in experimentally-infected sheep. Using this outbred animal model of B-cell transformation, oncogenic modifications reflected in altered microRNA expression can be identified and compared as the disease progresses. We have analyzed the miRNome of transformed B-cells isolated from leukemic sheep. Using Taqman Low Density Array (TLDA) assays and High-Throughput (HT) sequencing of small RNA libraries, we identified differentially-expressed microRNAs associated with B-cell transformation. For miRBase database-matched sheep orthologs there was a good overall quantitative correlation between data generated with both techniques. Furthermore, deep sequencing identified variants of mature microRNA transcripts, indicating that isomir distribution might be of biological significance. Finally, HT sequencing revealed unknown candidate microRNAs which were confirmed both in silico using miRDeep and experimentally using stem-loop RT-QPCR methods. Target prediction tools (miRanda, targetscan) suggest that these microRNAs might target both cellular and viral mRNAs. Down-regulation of viral mRNAs might contribute to tumor-associated virus silencing and play a role in immune escape mechanisms. Ongoing work aims at the validation of bioinformatics predictions of microRNA targets. Altogether, this work should lead to a better understanding of the microRNA-mRNA regulation network associated with leukemia progression.

